# Deep peroneal nerve injury during plate fixation for medial open‐wedge high tibial osteotomy: A case report and cadaveric study

**DOI:** 10.1002/ccr3.2451

**Published:** 2019-10-09

**Authors:** Masafumi Itoh, Junya Itou, Umito Kuwashima, Hiroki Fujieda, Ken Okazaki

**Affiliations:** ^1^ Department of Orthopedic surgery Tokyo Women's Medical University Tokyo Japan; ^2^ Department of Anatomy Tokyo Women's Medical University Tokyo Japan

**Keywords:** neurovascular injury, open‐wedge high tibial osteotomy, osteoarthritis, surgical complications

## Abstract

This report presents a case of deep peroneal nerve palsy caused by an injury during drilling for distal locking screws of a T‐shaped locking plate used for osteosynthesis of medial open‐wedge high tibial osteotomy. A cadaveric simulation study reproduced the risk of injury during the surgery.

## Abstract 2

Surgeons should acknowledge the hidden risk of injury of the deep peroneal nerve when drilling for the distal locking screws during medial open‐wedge high tibial osteotomy and avoid drilling too deeply after penetrating the lateral cortex or consider a monocortical fixation fashion.

## Graphical abstract

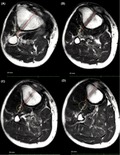

## 1 INTRODUCTION

Medial open‐wedge high tibial osteotomy (MOWHTO) is a promising surgical option for medial gonarthritis,^1^ which recently gained popularity after utilization of locking plates to provide rigid fixation, allowing weight bearing shortly after surgery.^2^, ^3^, ^4^, ^5^ MOWHTO‐related complications involving neurovascular injury are relatively rare compared with those related to lateral closed‐wedge high tibial osteotomy, which requires exposure of the lateral tibia and a second osteotomy for the fibula, potentially causing peroneal nerve injury.^1^, ^6^ Although a case of tibial nerve neuropathy following MOWHTO has previously reported,^7^ the present study describes the first case of deep peroneal nerve (DPN) palsy following MOWHTO. Magnetic resonance imaging (MRI) after plate removal revealed that the extended trajectory of the drilling holes for the locking plate's distal screws intersected the neurovascular bundle of the DPN, suggesting that DPN injury occurred during drilling. Cadaveric dissection confirmed the potential risk of DPN injury in a similar procedure.

## 2 CASE REPORT

**Figure 1:**
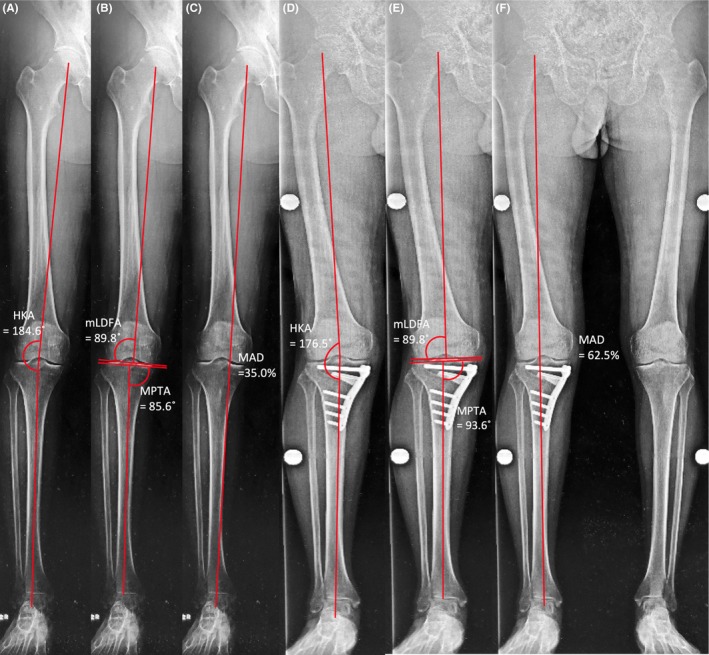
Preoperative (A, B, C) and postoperative (D, E, F) whole‐leg standing radiographs. The hip‐knee‐ankle angle (HKA) was corrected from varus 4.6° (A) to valgus 3.5° (D). The preoperative mechanical lateral distal femoral angle (mLDFA) and medial proximal tibial angle (MPTA) were 89.8° and 85.6°, respectively, and the postoperative mLDFA and MPTA were 89.8° and 93.6°, respectively (B, E). The mechanical axis deviation (MAD) was 35.0% from the medial edge of the tibial plateau preoperatively (C) and 62.5% postoperatively (F)

**Figure 2:**
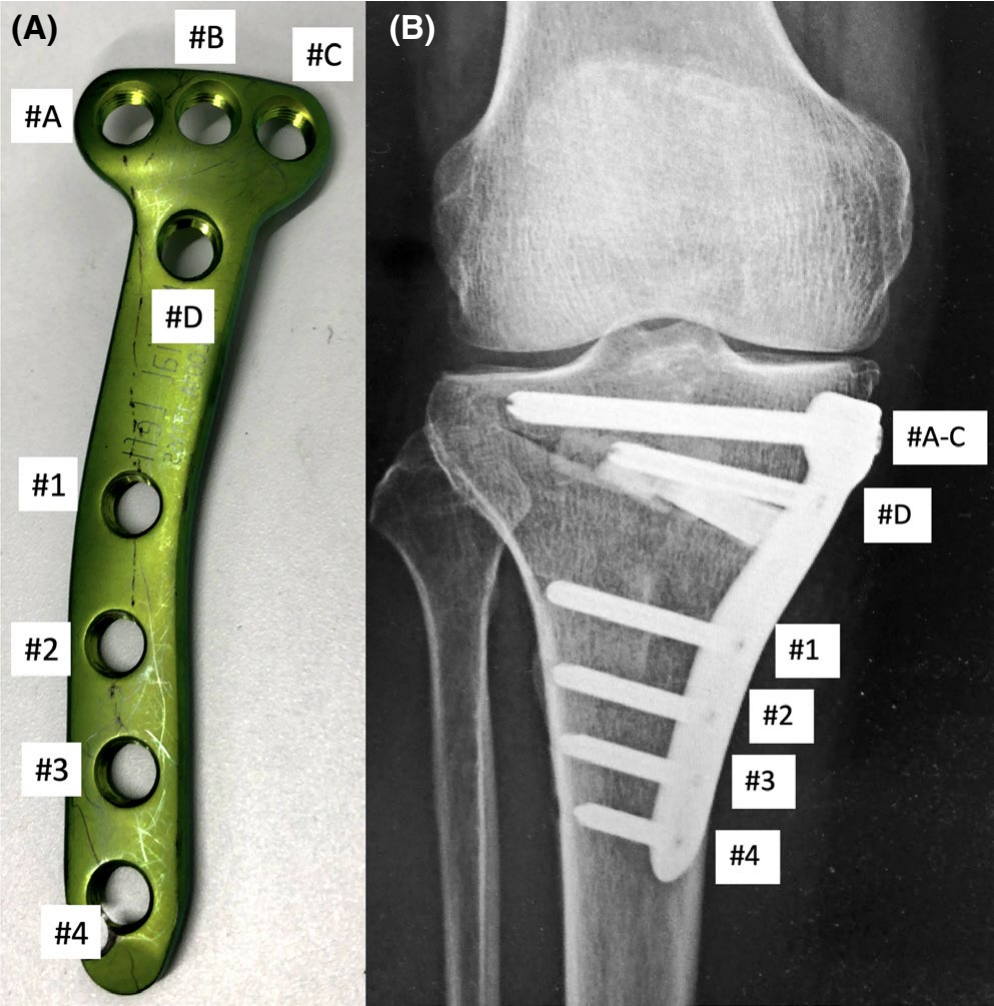
A, Tris medial HTO plate has screw holes #A–#D above the osteotomy site and holes #1–#4 below the osteotomy site. B, Screws #1–#3 were fixed bicortically; screw #4 was fixed monocortically

A 63‐year‐old Japanese man with an office job presented with severe pain in his right knee, which started approximately one year before. The right knee's range of motion was not restricted, and the medial joint space was tender. He presented with no symptom other than that in the right knee and did not have any medical and family history. A standing anterior‐posterior radiographic view revealed narrowing of the medial joint space, and preoperative MRI showed a horizontal tear in the posterior part of the medial meniscus, chondral wear of the medial compartment, and normal findings in the cruciate ligaments and lateral and patellofemoral compartment. A whole‐leg standing radiograph showed varus limb alignment with 4.6° hip‐knee‐ankle angle (HKA). The parameters described by Paley^8^ such as mechanical lateral distal femoral angle (mLDFA) and medial proximal tibial angle (MPTA), and mechanical axis deviation (MAD) were 83.8°, 85.6°, and 35.0%, respectively (Figure 1A, 1, C). During arthroscopic examination, a horizontal tear in the medial meniscus was trimmed. MOWHTO was performed as follows: the pedes anserini were detached from their insertion, and the superficial layer of the medial collateral ligament was released from the tibia. The soft tissues behind the tibia were posteriorly retracted to protect the posterior neurovascular structure. The osteotomy starting point was the upper border of the pes anserinus, aiming to reach the proximal third of the tibiofibular joint. The posterior osteotomy was stopped 10 mm before the lateral cortex. Ascending osteotomy was performed behind the tibial tuberosity to complete the biplanar osteotomy. Once the limb alignment was corrected by opening the medial side of the osteotomy, beta‐tricalcium phosphate blocks (OSferion 60, Olympus Terumo Biomaterials Corp.) were inserted into the osteotomy gap. Osteosynthesis was performed using a T‐shaped long locking plate (TriS medial HTO plate; Olympus Terumo Biomaterials Corp), which comprised a total of eight screws, four for proximal fixation and four for distal fixation (Figure 2A). Among the distal fixation screws, three proximal ones (ie screws #1‐3) were bicortically fixed, whereas the most distal screw (ie screw #4) was monocortically fixed (Figure 2B). The length of the screw holes #1‐3 was measured using a depth gauge, which had a smooth tip, with a 2 × 1 mm size. The HKA, MPTA, and MAD were corrected to anatomical valgus 3.5°, 93.6°, and 62.5%, respectively (Figure 1D, 1, F).

**Figure 3:**
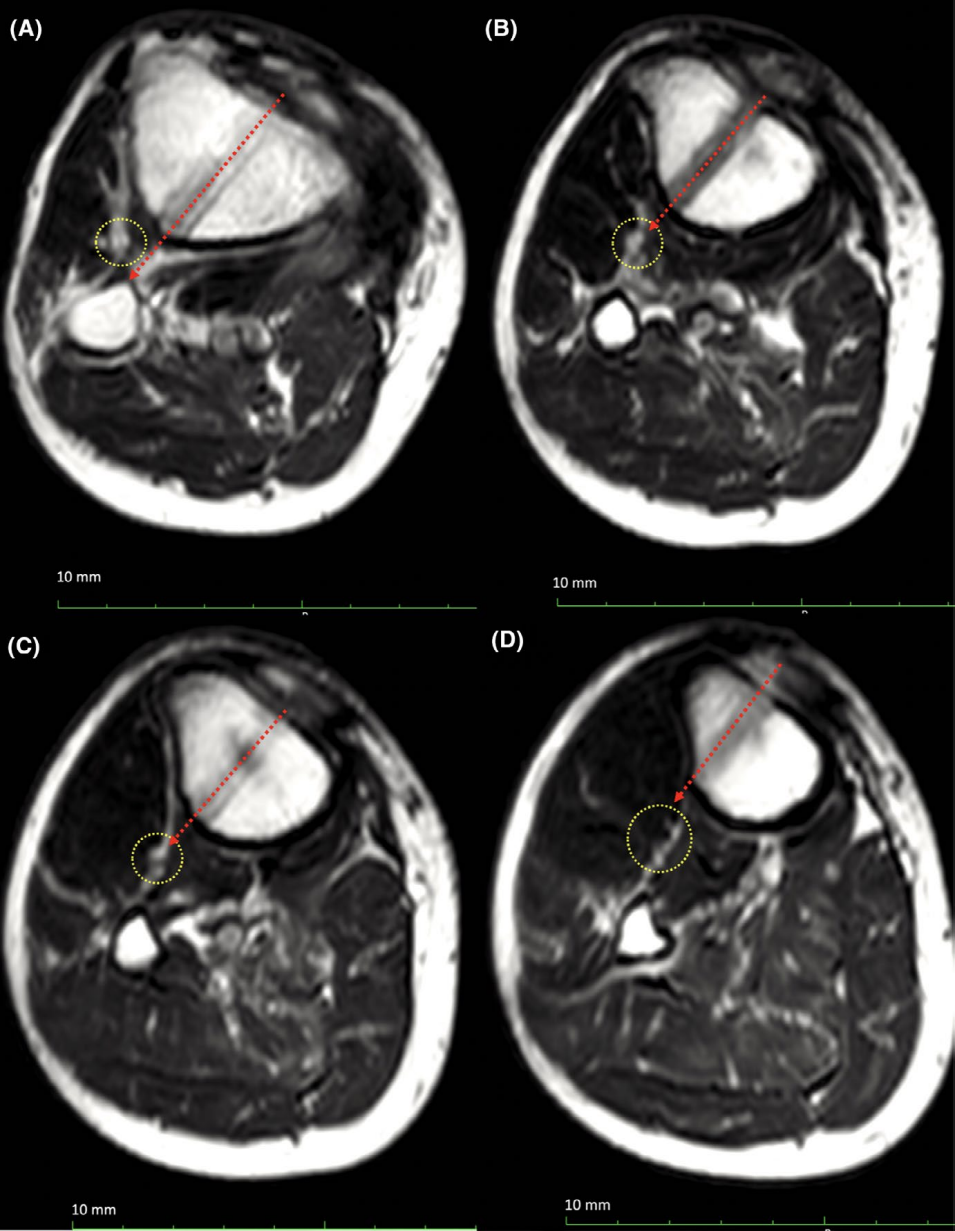
Magnetic resonance imaging at the level of distal locking screw holes (A, #1; B, #2; C, #3; D, #4) indicating the location of the neurovascular bundle including the deep peroneal nerve (yellow dotted circle) and direction of drilling for screw hole (red dotted arrow)

Immediately after surgery, the patient was unable to extend the right great toe and complained of numbness in the dominant area of the right DPN. The patient was diagnosed with palsy of the DPN branch supplying the extensor hallucis longus (EHL). A nerve conduction velocity test performed four months after surgery showed an amplitude decline in the DPN‐innervated muscles. The patient preferred not to undergo further surgery, such as nerve exploration, because loss of the right great toe dorsiflexion was not a very severe problem for him. Ten months postoperatively, the Tris medial HTO plate was removed with confirmed bone union due to irritation caused by the plate. MRI after plate removal revealed that the extended trajectories of the distal locking screw holes were close to the DPN‐containing neurovascular bundle (Figure 3). On the lateral surface of the tibia, the distances between the neurovascular bundle and outlet of the drill hole were 8 and 9 mm for screws #2 and #3, respectively. The distance was only 6 mm for screw #4, while it was fixed monocortically in this case. Although, from seven months postoperatively, EHL muscle weakness had partially recovered, an electromyogram test performed 15 months postoperatively showed fibrillation potential and positive sharp wave, each known as denervation potential only in EHL (Table 1). It was suggested that the DPN branch that innervates the EHL was severely injured. The relevant physical examination and clinical findings of this case are summarized in a timeline (Table 2). The patient provided informed consent for publication of this case.

**Table 1:** Electromyogram findings 15 mo postoperatively

Muscle	Side	Fibrillation potential	Positive sharp wave
EDL	Right	0	0
EHL	Left	0	0
EHL	Right	+1	+3
PL	Left	0	0
PL	Right	0	0
TA	Right	0	0

Abbreviations: DPN, deep peroneal nerve; EDL, extensor digitorum longus; EHL, extensor hallucis longus; PL, peroneus longus; TA, tibialis anterior.

**Table 2:** Timeline of the clinical case

Timeline (postoperative)	Event
0 d	Surgery
0 d	Dysfunction of the right great toe
4 mo	Nerve conduction velocity test
7 mo	Partial recovery of right great toe strength
10 mo	Plate removal
11 mo	Magnetic resonance imaging of the lower thigh
15 mo	Electromyogram of the lower thigh

### 2.1 Cadaveric study

**Figure 4:**
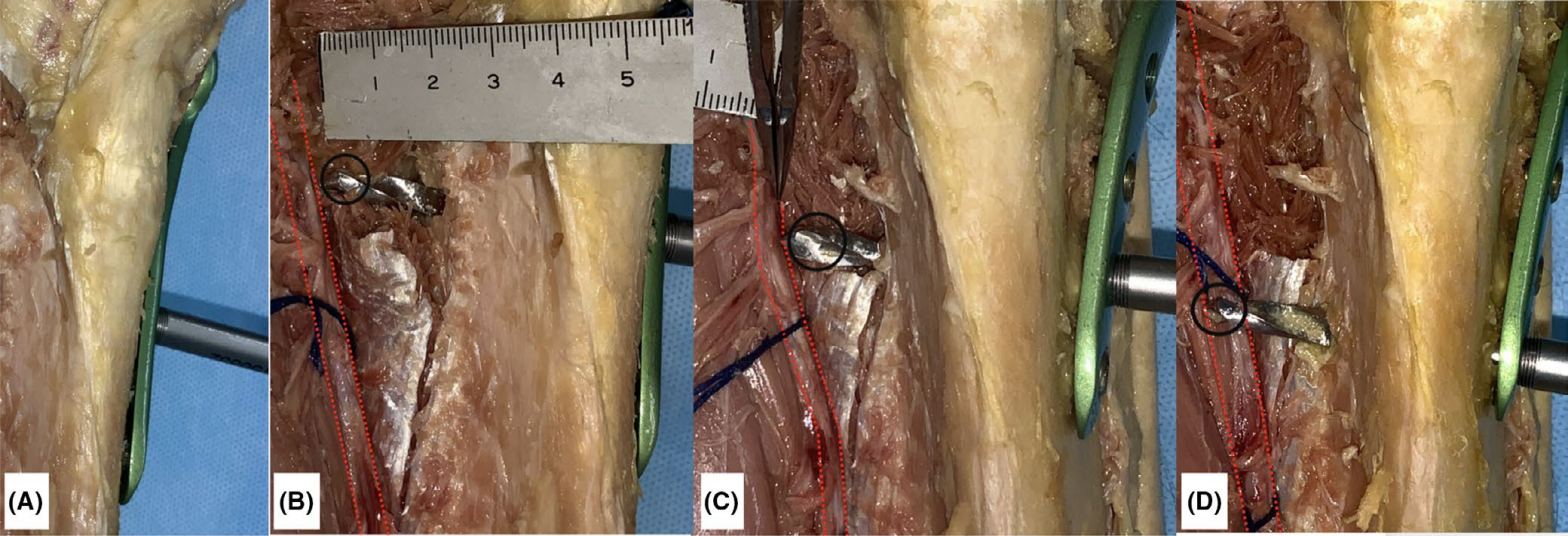
Open‐wedge high tibial osteotomy was simulated in a cadaveric knee using TriS medial HTO plate (A). The drill tip (black circle) penetrated the neurovascular bundle containing the deep peroneal nerve (red dotted line). B, Drilling through hole #2. C, Drilling through hole #3. D, Drilling through hole #4

To identify the positional relationship between the DPN‐containing neurovascular bundle and the upper third of the tibia, a cadaveric right lower leg was dissected. During MOWHTO, osteosynthesis was simulated using the Tris medial HTO plate. The DPN‐containing neurovascular bundle was located only about 10‐15 mm away from the tibia. The drill tip through the holes of screws #2 and #3 penetrated the neurovascular bundle, and the drill tip through the holes of screws #4 scratched the neurovascular bundle and then passed in front of the bundle (Figure 4A‐D). The study protocol was reviewed and approved by the institutional review board (approval number, 5044).

## 3 DISCUSSION

To the best of the author's knowledge, this is the first case report on DPN palsy following MOWHTO. Since DPN does not branch from the common peroneal nerve at the osteotomy site level, it cannot be injured during osteotomy. Therefore, DPN injury caused by drilling through the distal locking screw holes seemed to be the reason for the development of DPN palsy immediately after surgery. MRI performed after plate removal revealed that the extended trajectory of the distal locking screws’ drill holes passed close to the DPN‐containing neurovascular bundle. The distance between the neurovascular bundle and the screw hole outlets was only about 5‐9 mm. Because, in this case, screw #4 was fixed monocortically, it was suggested that the DPN was injured by drilling through screw hole #2 or #3.

Few cases of nerve injury following MOWHTO have been reported. Shin et al reported a case of tibial nerve neuropathy during MOWHTO, which was caused by placement of an excessively long screw through the screw hole just below the osteotomy site.^7^ Tibial nerve and popliteal vessels are located only 0.5‐1 cm behind the tibia, at the tibial osteotomy level. Since both the popliteus and tibialis posterior muscles are located between the tibia and neurovascular structure, the tibial nerve and popliteal vessels are at risk of injury during tibial osteotomy, unless retractors are correctly placed in the space between the popliteus muscle and tibia.^9^ Neurovascular injury can occur during osteotomy and screw fixation. In the study by Shin et al, the plate was anteriorly positioned, posing a risk for the screw to penetrate the tibial nerve.^7^ It is recommended to medially position the locking plates as much as possible to maximize fixation strength and avoid the risk of injury to the popliteal vessels and tibial nerve.^10^, ^11^ However, the risk of DPN injury by drilling of the distal locking screws has not been recognized. We also underestimated the situation of this patient and considered EHL dysfunction as temporal and mild. This was the reason that we performed nerve conduction studies remarkably late, four months postoperatively.

Kirgis et al performed a cadaveric study using 29 specimens and reported that 91% of the DPN motor branches supplying the EHL branched from the trunk of the DPN 68‐136 mm distal to the fibular head, and 83% penetrated the EHL 5‐28 mm below the bifurcation point.^12^ They suggested that fibular or tibial osteotomy from 68 to 153 mm distal to the fibula head may lead to EHL dysfunction. Since the length of the Tris medial HTO plate is 110 mm, the screw holes for distal fixation are located within this area. The TomoFix Medial High Tibial Plate, which is widely used worldwide, also has screw holes within this area. MRI of our case revealed that the DPN‐containing neurovascular bundle was located <10 mm anterior to the screw hole. Additionally, surgical simulation in a cadaver confirmed the risk of injury during drilling of the screw hole. Furthermore, measuring screw hole length using a depth gauge also leads to a risk of DPN injury. Although the tip of the depth gauge is smooth, a blind procedure in measuring the screw hole length using the depth gauge may lead to a risk of injury. Therefore, meticulous attention should be paid not to penetrate the lateral cortex too deeply not only when drilling but also in using the depth gauge. It is considered to be a common hidden risk during MOWHTO, and this case is most likely not the only one. Several cases have reported DPN injury by proximal interlocking screws from the anteromedial to posterolateral direction of the intramedullary nail,^13^ through an injury mechanism similar to the one presented here. Although there have been no previous studies on DPN injury following MOWHTO, its incidence may be underestimated since the degree of disability is milder than that of the common peroneal nerve or tibial nerve palsy. In fact, the patient in the present case showed gradual recovery from DPN palsy seven months postoperatively, and electromyogram displayed a high amplitude wave in EHL. It was suggested that the drill bit penetrated and injured the DPN but, fortunately, did not completely cut it.

In MOWHTO, DPN has a risk of injury during drilling for distal locking screws. This risk can be minimized by avoiding drilling too deeply beyond 10 mm, after penetrating the lateral cortex wall. Moreover, a monocortical fixation method can be used. However, even though screw #4 was monocortically fixed, in the present case, the DPN was injured through the other screw holes. Therefore, surgeons should acknowledge a similar risk for other screw holes. One of the advantages of the locking plate system is that it provides more rigid fixation than the nonlocking plate system when utilizing the monocortical fixation method. Monocortical screw fixation can be considered for other screws as long as the fixation stability is secured. Further biomechanical investigation should be conducted to determine how many of the four screws can be fixed monocortically to obtain rigid fixation.

## CONFLICT OF INTEREST

None.

## AUTHOR CONTRIBUTIONS

Itoh M involved in primary care physician of this case, cadaveric study, and manuscript preparation. Itou J involved in secondary care physician of this case and cadaveric study. Kuwashima U participated in cadaveric study and manuscript preparation. Fujieda H participated in cadaveric study authorization and conduct. Okazaki K helped in supervision of the care of this case, presentation, and manuscript preparation.
